# A Multi-Objective Optimization Framework That Incorporates Interpretable CatBoost and Modified Slime Mould Algorithm to Resolve Boiler Combustion Optimization Problem

**DOI:** 10.3390/biomimetics9110717

**Published:** 2024-11-20

**Authors:** Shan Gao, Yunpeng Ma

**Affiliations:** School of Information Engineering, Tianjin University of Commerce, Beichen, Tianjin 300134, China; gaoshan@tjcu.edu.cn

**Keywords:** multi-objective optimization framework, boiler combustion optimization, interpretable CatBoost, slime mould algorithm

## Abstract

The combustion optimization problem of the circulation fluidized bed boiler is regarded as a difficult multi-objective optimization problem that requires simultaneously improving the boiler thermal efficiency and reducing the NOx emissions concentration. In order to solve the above-mentioned problem, a new multi-objective optimization framework that incorporates an interpretable CatBoost model and modified slime mould algorithm is proposed. Firstly, the interpretable CatBoost model combined with TreeSHAP is applied to model the boiler thermal efficiency and NOx emissions concentration. Simultaneously, data correlation analysis is conducted based on the established models. Finally, a kind of modified slime mould algorithm is proposed and used to optimize the adjustable operation parameters of one 330 MW circulation fluidized bed boiler. The experimental results show that the proposed framework can effectively improve the boiler thermal efficiency and reduce the NOx emissions concentration, where the average optimization ratio for thermal efficiency reaches +0.68%, the average optimization ratio for NOx emission concentration reaches −37.55%, and the average optimization time is 6.40 s. In addition, the superiority of the proposed method is demonstrated by ten benchmark testing functions and two constrained optimization problems. Therefore, the proposed framework is an effective artificial intelligence approach for the modeling and optimization of complex systems.

## 1. Introduction

### 1.1. Background

At present, thermal power generation still dominates the supply of electric energy, and coal is the main thermal power supply resource. However, as a kind of non-renewable energy source, coal is becoming more and more scarce with the massive consumption of thermal power generation. And the combustion process will be accompanied by large amounts of pollutants discharged into the atmosphere, such as NOx and SO_2_, which seriously affect the atmospheric environment and human living. Therefore, it is an urgent problem to improve boiler’s thermal efficiency and reduce NOx emissions for thermal power generation.

The above-mentioned problem is regarded as a multi-objective optimization problem, which is called a boiler combustion optimization problem, and it is also an important research direction [1]. It is widely known that the boiler combustion system has complex characteristics with non-linearity, strong coupling and large hysteresis, making it difficult to be optimized by traditional mathematical and statistical methods. To address this problem, many researchers commonly used artificial neural networks to build the thermal efficiency model or a NOx emissions model based on the boiler’s historical operation data [2,3,4,5]. And based on the established model, all kinds of optimization algorithms were applied to adjust the running parameters for improving the thermal efficiency and reducing NOx emissions. In addition, solving the optimization problem of the boiler combustion process is beneficial to the ecological modernization of power plants [6,7].

For modeling, Zhou et al. [8,9,10] applied artificial neural networks (ANNs) and support vector machine (SVM) variants to develop the prediction models for thermal efficiency and NOx emission concentration. The experimental results showed that ANNs or SVM variants can obtain good prediction results. These research results had an important impact on the subsequent research on the boiler combustion optimization problem. Si et al. [11] applied an improved SVM to achieve a dynamic adaptive model of NOx emission concentration, which provided a new idea for online modeling. Li et al. applied the extreme learning machine (ELM) and fast learning network (FLN) to model thermal efficiency and NOx emission [12,13,14]. The experimental results showed that the ELM and FLN can obtain higher accuracy and faster computing speed than ANNs and SVMs. Ma et al. [15] proposed a hyper-parameter self-optimized broad learning system to model the thermal efficiency and emission concentrations of Nox and SO_2_.

For optimization, researchers were keen on applying meta-heuristic optimization algorithms to solve the boiler combustion optimization problem [16,17,18]. Rahat et al. [19] applied a kind of novel multi-objective optimization algorithm to solve the contradiction between thermal efficiency and NOx emission concentration. Wang et al. [20] combined random forest (RF) with an improved flower pollination algorithm to build a NOx emission concentration model and reduce NOx emissions, separately. Hu et al. [21] used a deep neural network and multi-objective optimization algorithm to balance boiler thermal efficiency and NOx emission concentration. Three years ago, Xu et al. proposed several methods to solve the boiler combustion optimization problem, including the ICFAR-IPSO-LSTM-IMOPSO model [22], CIPSODM-LSTM algorithm framework [23], data-driven evolutionary optimization and online retrieval method [24]. In our group, Li and Ma et al. [25,26,27] used an improved artificial bee colony (ABC) and teaching and learning optimization (TLBO) to enhance the thermal efficiency and reduce the NOx emission concentration. In summary, artificial neural networks and meta-heuristic optimization algorithms can effectively solve the boiler combustion optimization problem.

### 1.2. Motivation

In this paper, a new multi-objective optimization framework is proposed to solve the boiler combustion optimization problem. The proposed framework combines the interpretable CatBoost model by TreeSHAP and a modified slime mould algorithm (MSMA). The interpretable CatBoost model is firstly used to build the synthetic model of boiler thermal efficiency and NOx emissions based on the historical operation data of one 330 MW circulation fluidized bed boiler (CFBB). Based on the established synthetic model, the modified slime mould algorithm is used to optimize the CFBB’s adjustment operation parameters for improved thermal efficiency and reduced NOx emissions. The experimental results show that the interpretable CatBoost model can obtain better model performance than RF [28,29], SVMs [30,31] and MLP [32,33,34], and the MSMA can improve boiler thermal efficiency and reduce NOx emissions.

Now, the new proposed framework needs to be explained. For the model method, CatBoost [35,36,37] is a kind of novel ensemble learning model that was first proposed by Yandex in 2017, and TreeSHAP [38,39] is an interpretable method for black box models that was proposed by Lundberg in 2017. Combining CatBoost with TreeSHAP can enhance its interpretability, which is called the interpretable CatBoost model in this paper. For the optimization algorithm, a slime mould algorithm (SMA) [40] is a kind of novel meta-heuristic algorithm that was first proposed by Lee et al. in 2020, which mainly simulates the changes of behavior and morphology of slime mould in nature during foraging. The SMA has several advantages, including a simple principle, few parameters and good global search capability. In order to further enhance the convergence accuracy and convergence speed of the original SMA, two mechanisms are proposed and introduced in the SMA. Firstly, iterative chaotic mapping with infinite collapse (ICMIC) [41] is used to initialize the population individuals for increasing population diversity and enhancing global search capability. Secondly, a reverse interpolation strategy (RIS) is proposed on the basis of opposition-based learning (OBL) [42,43] to update the positions of the population individuals obtained from each iteration, which helps to improve the quality of the solution and strengthen the local search ability. Compared with five other state-of-the-art meta-heuristic algorithms (including the butterfly optimization algorithm (BOA) [44], whale optimization algorithm (WOA) [45], seagull optimization algorithm (SOA) [46] and sparrow search algorithm (SSA) [47]) on 10 benchmark testing functions and two classical constrained optimization problems, the proposed MSMA outperforms on most testing functions. Moreover, compared to the original SMA, the MSMA is able to obtain better or equivalent convergence accuracy with less convergence time without increasing the calculation burden.

### 1.3. Contributions

In order to solve the boiler combustion optimization problem, a new multi-objective optimization framework is firstly designed in this paper. The contributions of this paper are summarized as follows:

1. A kind of novel multi-objective optimization framework is proposed, which combines interpretable CatBoost by TreeSHAP and a modified slime mould algorithm. Particularly, this framework has model interpretability and adaptivity.
2. A kind of modified slime mould algorithm is proposed to solve benchmark testing functions and constrained optimization problems.

The structure of this paper is as follows: the study object and basic methods are introduced in Section 2; the modified SMA is presented in Section 3; Section 4 contains the application of the proposed framework to the boiler combustion optimization problem; and the conclusions are presented in Section 5.

## 2. Related Work

### 2.1. Study Object

**Figure 1:**
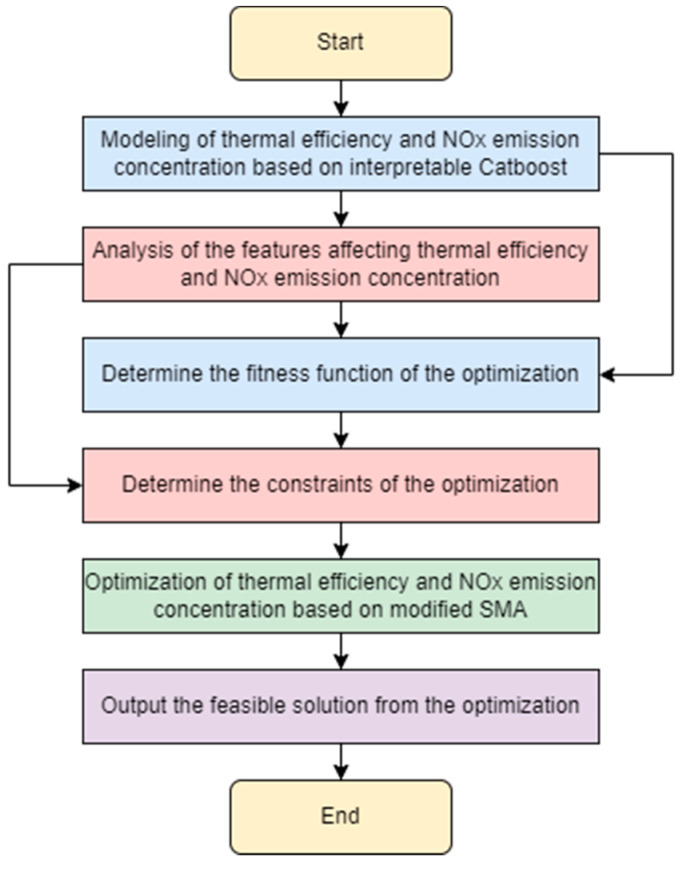
The flow chart of the proposed framework.

The study object of this paper is a 330 MW circulating fluidized bed boiler (CFBB). The main study focus of this paper is to maximize the thermal efficiency (TE) while minimizing the NOx emissions concentration of the CFBB. To achieve the optimization goal, a kind of new multi-objective optimization framework integrating the interpretable CatBoost model and MSMA is proposed in this paper. The flow chart of the proposed framework is shown in Figure 1, and the core steps are summarized as follows:

Step 1. The interpretable CatBoost model combined with TreeSHAP is used to build the synthetic model of boiler thermal efficiency and NOx emissions based on historical operation data. Note that the detailed descriptions of the interpretable CatBoost and TreeSHAP are given in the literature [35,36,37,38,39], separately.

Step 2. Based on the established model in step 1, the value ranges of adjustable parameters of the CFBB with positive impact on thermal efficiency and negative impact on NOx emissions are obtained by further analyzing the features affecting the thermal efficiency and NOx emission concentration.

Step 3. Based on the established model and selected parameter features, the boiler’s adjustment parameters are optimized by a modified SMA with the fitness function of maximizing the thermal efficiency and minimizing the NOx emission concentration of the CFBB. And the constraints for the value ranges of the adjustable parameters of the CFBB are obtained in step 2.

### 2.2. Slime Mould Algorithm (SMA)

In this subsection, a detailed descriptions of the slime mould algorithm is given. The specific content of the SMA is explained below. In order to understand the following method, the symbols used in this section are explained in Table 1.

The slime mould algorithm (SMA) is a kind of novel meta-heuristic algorithm that was first proposed by Lee et al. in 2020, which is inspired by foraging behavior and the morphology of slime mould. Slime mould is a eukaryotic organism that lives in a humid and cold environment and takes its nutrients mainly from external organic matter. When slime mould approaches its food source, its bio-oscillator generates a propagation wave through the vein to increase the flow of cytoplasm. The higher the concentration of food, the stronger the propagation wave generated by the bio-oscillator and the faster the cytoplasmic flow. The SMA has a simple principle, few adjustment parameters and strong global search capability. Therefore, the SMA is used to solve the boiler combustion optimization problem. Its basic principle is as follows.

(1) The stage of approaching food

**Figure 2:**
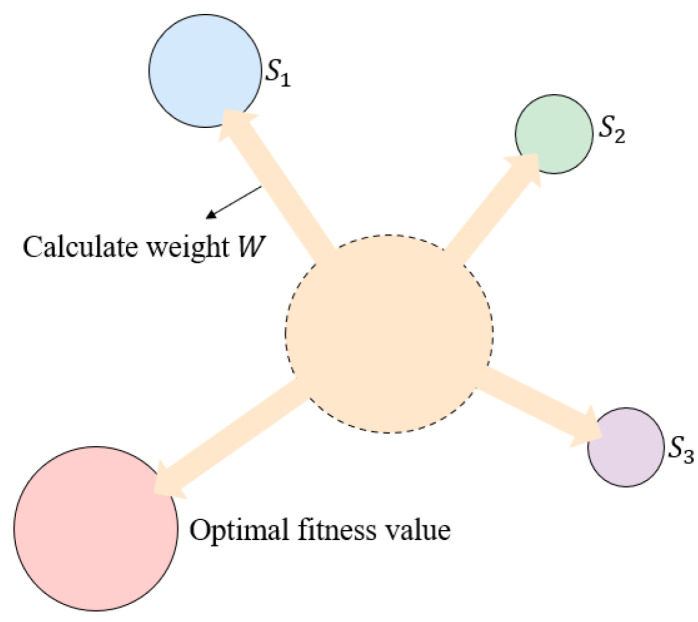
The fitness value evaluation graph of slime mould.

In the approaching food phase, the slime mould approaches the food through the odor in the air, and the expression of its approaching behavior is shown in Equation (1).

(1) $$X(t+1)=\left\{\begin{array}{c} X_{best}(t)+\mathrm{vb}\left(W\times X_{A}\left(t\right)-X_{B}\left(t\right)\right),\;r<q\\ vc\times X(t),\;r\geq q \end{array}\right.$$

where X(t+1) and X(t) are the positions of the slime mould individuals at the t+1th and tth iterations, respectively; $X_{best}(t)$ is the best position of the slime mould individual at the tth iteration; $X_{A}\left(t\right)$ and $X_{B}\left(t\right)$ are the two randomly selected slime mould individuals at the tth iteration; the range of vb is [−a,a], a=arctanh⁡(1−(t/P)), t is the current number of iterations and P is the total number of iterations; the range of vc is from 1 linearly decreasing to 0; r is a random value between 0 and 1; $q=tanh(\left|f(X_{i})-S\right|)$,i=1,2,…,Q, $f(X_{i})$ is the fitness value of the ith slime mould individual; S is the optimal fitness value in all iterations; Q is the population size of the slime mould individuals; and W is the weight of a slime mould individual, and its expression is shown in Equation (2).

(2) $$W(\mathrm{SortIndex}\;(X_{i}))=\left\{\begin{array}{c} 1+r\times \log\left(\frac{f_{best}-f(X_{i})}{f_{best}-f_{worst}}+1\right),\;condition\\ 1-r\times \log\left(\frac{f_{best}-f(X_{i})}{f_{best}-f_{worst}}+1\right),\;other \end{array}\right.$$

where condition is the top half of the population individual in terms of fitness value; $f_{best}$ and $f_{worst}$ are the best and worst fitness value at the current iteration, respectively; $SortIndex\;(X_{i})$ is the sequence of sorted fitness values; log⁡() is used to slow down the rate of change in the values and stabilize the change in the contraction frequency; condition and other are used to explain the process of slime mould adjusting its position according to the food concentration, where the higher the food concentration, the greater the weight of slime mould near the area; if the food concentration is low, slime mould will turn to search for other areas, and the weight of slime mould in the area will become smaller. The fitness value evaluation graph of slime mould is shown in Figure 2.

The approaching food stage simulates the positive and negative feedback process between the weight of slime mould and the food concentration. Equation (10) updates the individual positions according to the change in the optimal position $X_{best}$ and the fine tuning of vb, vc and W. The role of r is to form a search vector at any angle, i.e., to search the solution space in any direction, thus increasing the possibility of finding the optimal solution and better simulating the circular and fan-shaped structural motion of the slime mould as it approaches the food.

(2) The stage of enveloping food

The enveloping food phase simulates the contraction pattern of the slime mould venous tissue. The higher the concentration of food in contact with the venous tissue, the stronger the propagation wave generated by the bio-oscillator and the faster the cytoplasmic flow; the lower the concentration of food in contact with the venous tissue, the weaker the propagating waves generated by the biological oscillator and the slower the cytoplasmic flow. Based on the above principle, the expression of its enveloping behavior is shown in Equation (3).

(3) $$X(t+1)=\left\{\begin{array}{c} d\times (X_{ub}-X_{lb})+X_{lb},\;r<u\;\\ X_{best}(t)+vb\left(W\times X_{A}\left(t\right)-X_{B}\left(t\right)\right),\;r<q\\ vc\times X(t),\;r\geq q \end{array}\right.$$

where d is the random value generated within 0 to 1; $X_{ub}$ and $X_{lb}$ are the upper and lower bounds of the search space, respectively; and u is the proportion of randomly distributed slime mould individuals, and it is used to switch between the global search phase and the local search phase.

(3) The stage of grabbing food

During the food grasping phase, the slime mould uses the propagation waves generated by the bio-oscillator to change the velocity of cytoplasmic flow in the venous tissue, simulating the variation of the width of the venous tissue of slime mould with the variation of the oscillation frequency of the bio-oscillator by vb, vc and W. The slime mould approaches the food more slowly when the food concentration is low and more quickly when it finds good quality food. This stage is actually the update of the vb, vc and W.

## 3. Modified Slime Mould Algorithm (MSMA)

In order to further improve the convergence accuracy and convergence speed of the original SMA, a kind of modified slime mould algorithm (MSMA) is proposed by introducing two mechanisms. The detailed description of the two mechanisms is given in Section 3.1. In addition, ten benchmark testing functions and two classical constraint optimization problems are used to verify the performance of the MSMA in Section 3.2.

### 3.1. Improved Mechanism

(1) Iterative chaotic mapping with infinite collapse (ICMIC)

In swarm intelligence optimization algorithms, the quality of the initial population individuals has a large impact on the algorithm performance. A good initial population is conducive to obtaining a global optimal solution. The original SMA uses a random initialization method to initialize the population without a priori knowledge, which may easily lead to the population diversity missing. Logistic and tent chaotic mapping functions are commonly utilized to initialize population individuals for enhancing the population diversity. However, compared with these two chaotic mapping methods, the ICMIC chaotic mapping method has better uniform traversal and faster convergence. Therefore, the ICMIC chaos mapping method is introduced to generate population individuals.

The expression of the ICMIC chaotic mapping is shown in Equation (4).

(4) $$\left\{\begin{array}{c} Z_{i+1}=\sin\left(\frac{\beta \pi }{z_{i}}\right),\;\beta \in (0,+\infty )\\ -1\leq Z_{i}\leq 1,{\;Z}_{i}\neq 0 \end{array}\right.$$

where β is a random value that follows the normal distribution in the interval (0,+∞).

Next, the chaotic sequence individual is converted to the real solution space, and the conversion formula is shown in Equation (5).

(5) $$X_{i}=X_{lb}+\left(X_{ub}-X_{lb}\right)\cdot \frac{1+Z_{i}}{2}$$

where $x_{ub}$ and $x_{lb}$ are the upper and lower bounds of the search space, respectively; and $Z_{i}$ is the generated chaotic sequence.

(2) Reverse interpolation strategy (RIS)

In order to further enhance the local exploration capability of the original SMA, a reverse interpolation strategy (RIS) is proposed, which combines elite opposition-based learning and quadratic interpolation. The RIS makes it easy for population individuals to jump out of local optimal solutions.

Opposition-based learning (OBL) is an optimization mechanism that was first proposed by Tizhoosh in 2005 whose main principle is to compute and evaluate the inverse solution simultaneously for the current feasible solution and select the better solution as the next generation individual. Elite opposition-based learning (EOBL) expands the search space based on the former by exploiting the fact that elite individuals include richer effective information than general individuals.

Assuming that the elite individual of the slime mould population at the tth iterations (i.e., the current optimal solution) is $X_{best}=\left(x_{i1},x_{i2},\cdots ,x_{ij},\cdots ,x_{iM}\right)$, where M is the dimension of the search space, the expression of its elite opposition solution $X^{*}=\left(x_{i1}^{*},x_{i2}^{*},\cdots ,x_{ij}^{*},\cdots ,x_{iM}^{*}\right)$ is shown in Equation (6).

(6) $$X^{*}=\theta \left(X_{lb}+X_{ub}\right)-X_{best}$$

where θ is the random value that follows the normal distribution in the interval [0, 1]; $X_{ub}$ and $X_{lb}$ are the upper and lower bounds of the search space, respectively.

When the generated elite inverse solution exceeds the maximum boundary range of the search space, the out-of-bounds reset is performed using the random generation method, whose expression is shown in Equation (7).

(7) $$X^{*}=rand\left(X_{lb}+X_{ub}\right)$$

In order to further enhance the utility of EOBL and improve the global search capability of the algorithm, the quadratic interpolation method is introduced to further update the elite solutions obtained by EOBL.

Assuming that ${X_{A}}^{*}$ and ${X_{B}}^{*}$ are the two randomly selected slime mould individuals at the tth iteration and different from $X_{A}$ and $X_{B}$, then the fitness values of $X_{A}$, $X_{B}$ and elite opposition solution $X^{*}$ are $f({X_{A}}^{*})$, $f({X_{B}}^{*})$ and $f(X^{*})$. The expression of the solution after the quadratic interpolation $X^{**}=\left(x_{i1}^{**},x_{i2}^{**},\cdots ,x_{ij}^{**},\cdots ,x_{iD}^{**}\right)$ is shown in Equation (8).

(8) $$X^{**}=\frac{\left({X^{*}}^{2}-{{X_{B}}^{*}}^{2}\right)\times f({X_{A}}^{*})+\left({{X_{A}}^{*}}^{2}-{X^{*}}^{2}\right)\times f({X_{B}}^{*})+\left({{X_{B}}^{*}}^{2}-{{X_{A}}^{*}}^{2}\right)\times f(X^{*})}{2[\left(X^{*}-{X_{B}}^{*}\right)\times f({X_{A}}^{*})+\left({X_{A}}^{*}-X^{*}\right)\times f({X_{B}}^{*})+\left({X_{B}}^{*}-X_{A}\right)\times f(X^{*})]}$$

When the generated solution after quadratic interpolation is not as good as the elite inverse solution, it is selected according to the expression shown in Equation (9).

(9) $$X^{**}=\left\{\begin{array}{c} X^{*},\;f(X^{*})<f(X^{**})\\ X^{**}.\;f(X^{*})⩾f(X^{**}) \end{array}\right.$$

(3) Overview of MSMA

**Figure 3:**
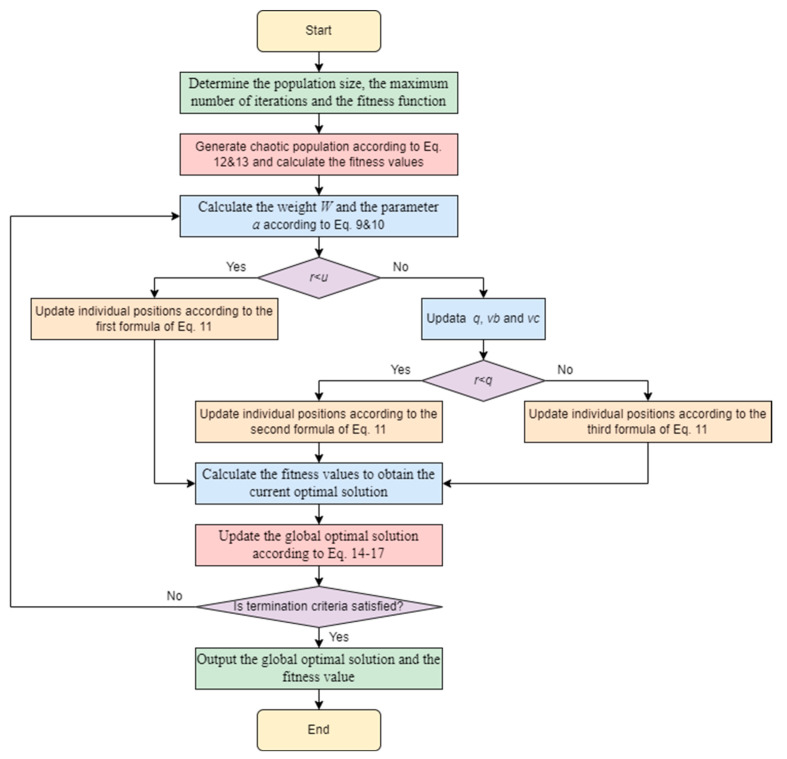
The flow chart of MSMA.

The flow chart of the MSMA is shown in Figure 3, and the specific steps are summarized as follows.

Step 1: Determine the population size, the maximum number of iterations and the fitness function.

Step 2: Generate chaotic initialized population individuals according to Equations (4) and (5) and calculate the fitness values.

Step 3: Calculate the weight W and the parameter a according to Equations (1) and (2).

Step 4: Generate random number r, and then judge the size of random value r and parameter u; if r<u, then update the individual position according to the first formula of Equation (3); otherwise, update parameters q, vb, vc. Judge the size of r and parameter q; if r<q, then update the individual position according to the second formula of Equation (3); otherwise, update the individual position according to the third formula of Equation (3).

Step 5: Calculate the fitness values to obtain the current optimal solution.

Step 6: Update the global optimal solution according to Equations (6)–(9).

Step 7: Determine whether the end condition is satisfied, and if so, output the global optimal solution and the fitness value; otherwise, repeat steps 2–6.

### 3.2. Experiments

In order to demonstrate the performance of the MSMA, ten benchmark testing functions are chosen from the CEC-2005 functions, and two classical constrained optimization problems are employed in this subsection. All the experiments including those in Section 4 are implemented on 11th Gen Intel(R) Core (TM) i7-11800H @ 2.30 GHz 2.30 GHz Processor and 32 GB RAM. All algorithms are coded and carried out in Python version 3.9 and Windows 11 Professional.

#### 3.2.1. Benchmark Testing Functions

The ten benchmark testing functions are shown in Table 2, where F1–F5 are single-peak testing functions and F6–F10 are multi-peak testing functions. The single-peak testing function has only one global optimal solution, which is mainly used to test the convergence speed and exploitation capability of the optimization algorithm. The multi-peak testing function has not only one global optimal solution, but also several local optimal solutions, which are mainly used to test the ability to jump out of the local optimal solution and the exploration capability of the optimization algorithm. In addition, five meta-heuristic algorithms are used to compare with the MSMA, including the original SMA [40], butterfly optimization algorithm (BOA) [44], whale optimization algorithm (WOA) [45], seagull optimization algorithm (SOA) [46] and sparrow search algorithm (SSA) [47]. According to references [40,44,45,46,47], parameters of the five algorithms are given in Table 3. For each testing function, the optimization algorithm runs independently 30 times. Three metrics are used to test the performance of the MSMA and other algorithms in terms of the mean, standard deviation (S.D.) and running time. The mean is the average of thirty optimum fitness values, and the running time is the average running time of thirty experiments. The population size of every algorithm is 50, and the maximum iteration number is 500. These testing functions are set to 30, 50 and 100 dimensions, separately. The experimental results are denoted from Table 4, Table 5 and Table 6.

**Figure 4:**
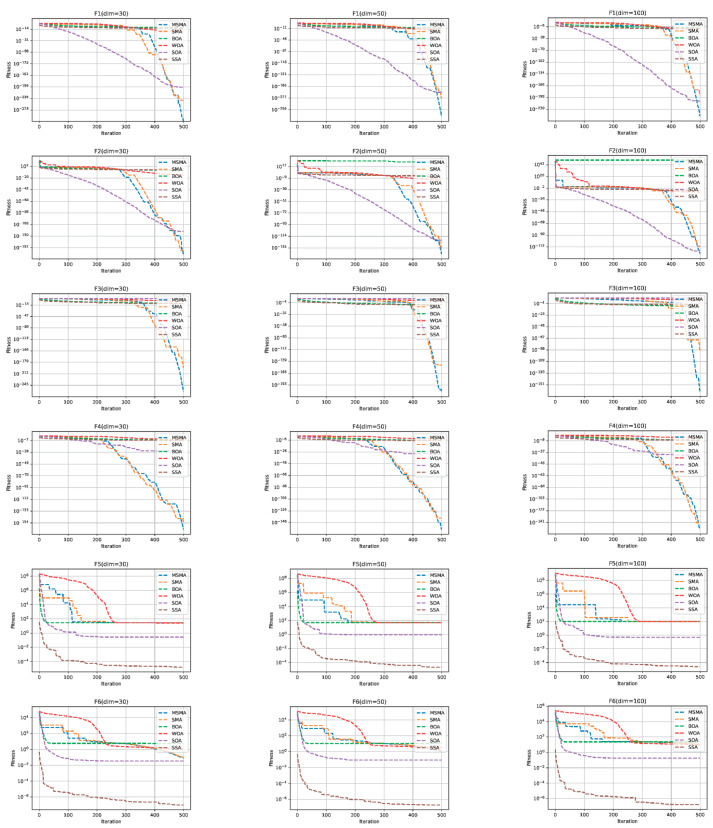

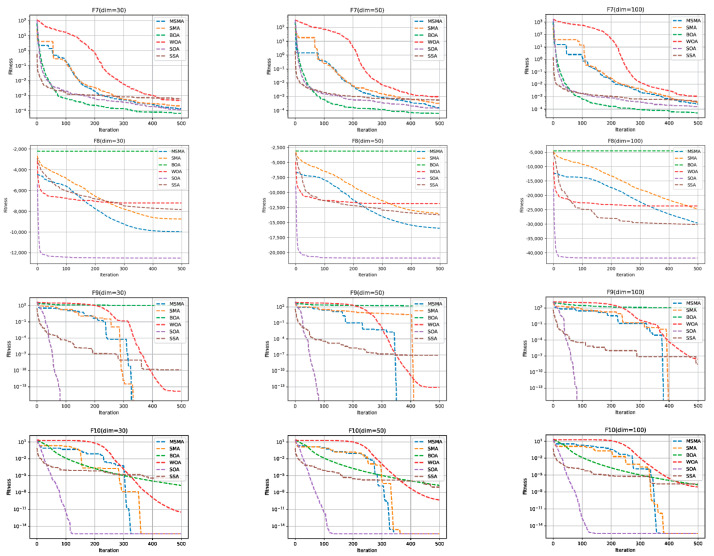
The convergence curves of six algorithms on 30, 50 and 100 dimensional functions.

The best optimal solutions in terms of the mean, standard deviation and running time are shown in boldface. And it is noted that the smaller the value of these statistics metrics, the better the performance of the optimization algorithm. In addition, the convergence curves of the MSMA and other algorithms are shown in Figure 4.

As can be seen from Table 4, Table 5 and Table 6 and Figure 4, the MSMA has the lowest mean and lowest standard deviation of all the methods on functions F1, F2, F3, F4, F9 and F10. The SSA has the smallest mean and standard deviation for functions F5 and F6. The BOA has the smallest mean and standard deviation for function F7. The SOA has the smallest mean and standard deviation for function F8. And the SOA has the shortest calculation time of all the algorithms for all functions. For the functions F5, F6, F7 and F8, although the MSMA does not obtain the best model performance, it outperforms the original SMA in both mean and standard deviation.

#### 3.2.2. Constrained Optimization Problem

(1) Optimal design of three-bar truss problem

In the optimal design of the three-bar truss problem, the variables $x_{1}$, $x_{2}$ and $x_{3}$ are the cross-sectional areas of the three bars, respectively, and by symmetry $x_{1}=x_{3}$. And the objective of the optimal design of the three-bar truss is to minimize the volume of the three-bar truss by adjusting the cross-sectional area ${(x}_{1},x_{2})$. This optimal design problem has a nonlinear fitness function, three nonlinear inequality constraints and two continuous decision variables, as shown in Equations (10)–(15).

Fitness function:

(10) $$minf(x)=\left(2\sqrt{2}x_{1}+x_{2}\right)100$$

Constraints:

(11) $$0.001\leq x_{1}\leq 1$$

(12) $$0.001\leq x_{2}\leq 1$$

(13) $$\frac{\sqrt{2}x_{1}+x_{2}}{\sqrt{2}x_{1}^{2}+2x_{1}x_{2}}2-2\leq 0$$

(14) $$\frac{x_{2}}{\left(\sqrt{2}x_{1}^{2}+2x_{1}x_{2}\right)}2-2\leq 0$$

(15) $$\frac{1}{\left(\sqrt{2}x_{2}+x_{1}\right)}2-2\leq 0$$

**Figure 5:**
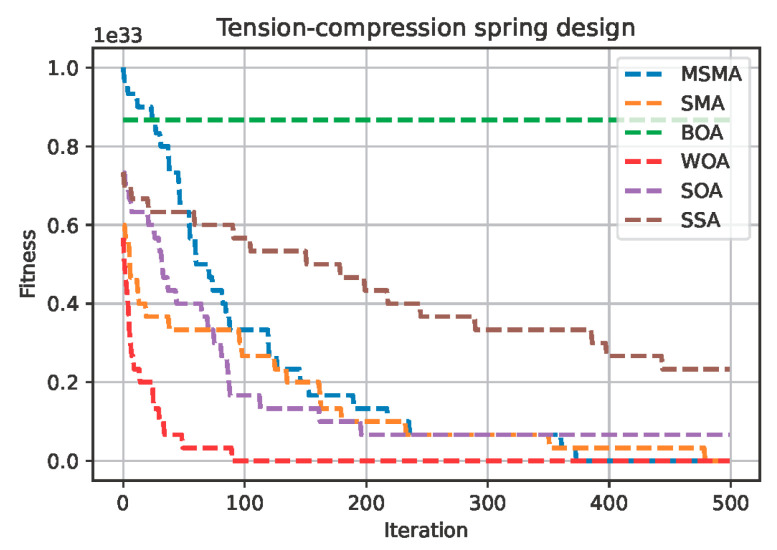
The convergence curve of six algorithms on the optimal design of three-bar truss problem.

The optimal solutions of the MSMA and the other algorithms used for comparison on the optimal design of the three-bar truss problem are shown in Table 7, and the convergence curve of six algorithms on this optimal design problem is shown in Figure 5.

As can be seen from Table 7 and Figure 5, the MSMA has the lowest mean and standard deviation of all the methods, and the WOA has the shortest running time.

(2) Optimal design of tension–compression spring problem

In the optimal design of the tension–compression spring problem, the variables $x_{1}$, $x_{2}$ and $x_{3}$ are the spring coil diameter, spring coil diameter and number of coils wound, respectively. The objective of the optimal design of the tension–compression spring is to minimize the weight of the tension–compression spring while meeting the minimum deflection, vibration frequency and shear stress. This optimal design problem has a nonlinear fitness function, four nonlinear inequality constraints and three continuous decision variables, as shown in Equations (16)–(23).

Fitness function:

(16) $$minf(x)=\left(x_{3}+2\right)x_{2}x_{1}^{2}$$

Constraints:

(17) $$0.05\leq x_{1}\leq 2$$

(18) $$0.25\leq x_{2}\leq 1.3$$

(19) $$2\leq x_{3}\leq 15$$

(20) $$1-\frac{x_{2}^{3}x_{3}}{71785x_{1}^{4}}\leq 0$$

(21) $$\frac{4x_{2}^{2}-x_{1}x_{2}}{12566\left(x_{2}x_{1}^{3}-x_{1}^{4}\right)}+\frac{1}{5108x_{1}^{2}}-1\leq 0$$

(22) $$1-\frac{140.45x_{1}}{x_{2}^{2}x_{3}}\leq 0$$

(23) $$\frac{x_{1}+x_{2}}{1.5}-1\leq 0$$

**Figure 6:**
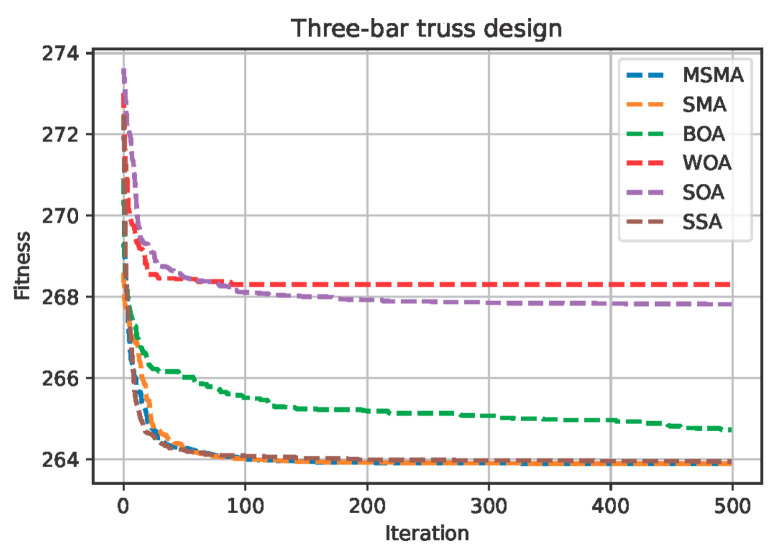
The convergence curve of six algorithms on the optimal design of tension–compression spring problem.

The optimal solutions of the MSMA and the other algorithms used for comparison on the optimal design of the tension–compression spring problem are shown in Table 8. And the convergence curves of six algorithms on this optimal design problem are shown in Figure 6.

As can be seen from Table 8 and Figure 6, the WOA has the lowest mean, lowest standard deviation and shortest calculation time of all the methods for this optimal design problem. Although the MSMA did not obtain the best model performance for this optimal design problem, it outperforms the original SMA on both the mean and standard deviation.

### 3.3. Discussion and Analysis

By solving the ten benchmark testing functions and two constrained optimization problems, the experimental results reveal that the MSMA is an effective meta-heuristic algorithm. The MSMA is good at handling the single-peaked function extremum problems, because it can obtain the optimal solution on four single-peaked functions F1, F2, F3 and F4. The MSMA needs to be further enhanced in handling multi-peaked function extremum problems because it can only obtain the optimum solution on two multi-peaked functions, F9 and F10. Although the MSMA did not obtain the optimal solution on functions F5, F6, F7, and F8, it outperformed the original SMA for all four functions. In addition, the MSMA also showed good performance on two constrained optimization problems.

The time complexity of calculating the SMA depends on the following aspects: population initialization of slime mould, evaluation and ranking of fitness values, weight update, and position update of slime mould individuals. Since the slime mould population size is Q, the research problem dimension is M, and the maximum number of iterations is P, the time complexity of the original SMA is O(M+P×Q×(1+logQ+M) according to the description of the SMA and the operation rules of time complexity. For the MSMA, the time complexity of ICMIC chaotic mapping to initialize the slime mould population is O(D), the time complexity of the fitness evaluation and ranking is O(Q+Q×logQ), the time complexity of the weight update is O(Q×M), the time complexity of the position update of slime mould individuals is O(Q×M), and the reverse interpolation strategy to generate new positions is O(Q×M); then, the overall time complexity of the MSMA is O(M+P×Q×(1+logQ+M). The time complexity of the MSMA is the same as that of the original SMA, indicating that the improved algorithm does not increase the computational burden.

Overall, the MSMA is more effective than the original SMA, and it can obtain better or equivalent convergence accuracy with fewer convergence times without adding much calculation time.

## 4. Boiler Combustion Optimization

In this section, the proposed multi-objective optimization framework is used to solve the boiler combustion optimization problem. Firstly, the description of boiler operation data is given in Section 4.1. Secondly, the interpretable CatBoost is applied to model the boiler thermal efficiency and NOx emissions concentration in Section 4.2. Based on the established model, data correlation analysis is conducted, determining the range of the parameters to be optimized. In Section 4.4, the proposed MSMA is used to optimize these adjustment parameters for improving thermal efficiency and reducing NOx emission concentration at the same time.

### 4.1. The Source and Description of Boiler Data

There are mainly 26 input data samples that affect the thermal efficiency (TE) and nitrogen oxide (NOx) emission concentration of the CFBB, including the boiler load, coal feed rate, average bed temperature, air flow, air temperature, etc. The symbols and descriptions of each sample are shown in Table 9. A total of 2880 sets of data were collected from the DCS of the CFBB. Some of these data are shown in Table 10.

### 4.2. Modeled by Interpretable CatBoost

In this subsection, the interpretable CatBoost model is used to build a comprehensive model of the boiler thermal efficiency and NOx emissions concentration. In order to evaluate the model superiority of the interpretable CatBoost model, three state-of-the-art methods are employed to compare the model accuracy and generalization capability, including random forest (RF), support vector machine (SVM), and multi-layer perceptron (MLP). The parameter settings of each method are given in Table 11. Four metrics are used to verify the performance of each method, such as root mean square error (RMSE), mean absolute error (MAE), mean absolute percentage error (MAPE) and goodness of fit (R^2^) of ten-fold cross-validation. For training data, the smaller the parameters (RMSE, MAE and MAPE) and the closer the R^2^ value is to one, the higher the model accuracy. For testing data, the smaller the parameters (RMSE, MAE and MAPE) and the closer the R^2^ value is to one, the better the generalization capability.

The data shown in Table 10 were normalized. Then, the total 2880 sets of data were divided into training data and testing data in a ratio of 7:3 according to the rule of ten-fold cross-validation. The experimental results are shown in Table 12.

As shown in Table 12, compared with RF, SVM and MLP, the interpretable CatBoost model can obtain better model accuracy and adequate generalization capability in the feature models established for the thermal efficiency and NOx emissions concentration of the CFBB both on the training set and testing set.

**Figure 7:**
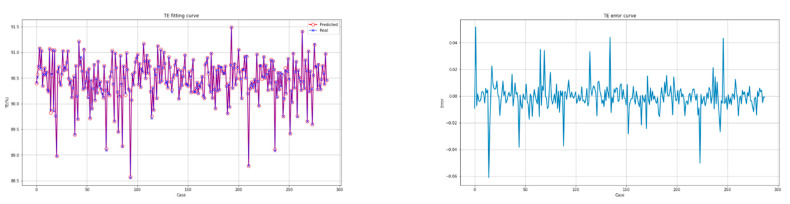
The fitting diagram and error diagram of the interpretable CatBoost model for modeling the thermal efficiency.

**Figure 8:**
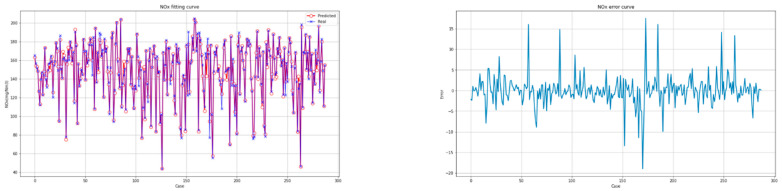
The fitting diagram and error diagram of the interpretable CatBoost model for modeling the NOx emissions concentration.

The fitting diagrams and error diagrams of the interpretable CatBoost model for modeling the two objectives of the CFBB on the partial testing set are shown in Figure 7 and Figure 8, where the red line and the blue line in the fitting diagram indicate the predicted values and real values of the partial testing set, and the light blue line in the error diagram represents the error of the predicted values compared to the real values for the partial testing set.

As shown in Figure 7 and Figure 8, the interpretable CatBoost model can effectively establish the feature models for the two objectives of the CFBB. The two feature models all have a great fitting effect and small fitting error on the testing set.

### 4.3. Data Feature Analysis

In response to the weak interpretation of CatBoost, an interpretable method combined TreeSHAP is applied to investigate the effect of the combustion condition of the CFBB on the thermal efficiency and NOx emission concentration.

**Figure 9:**
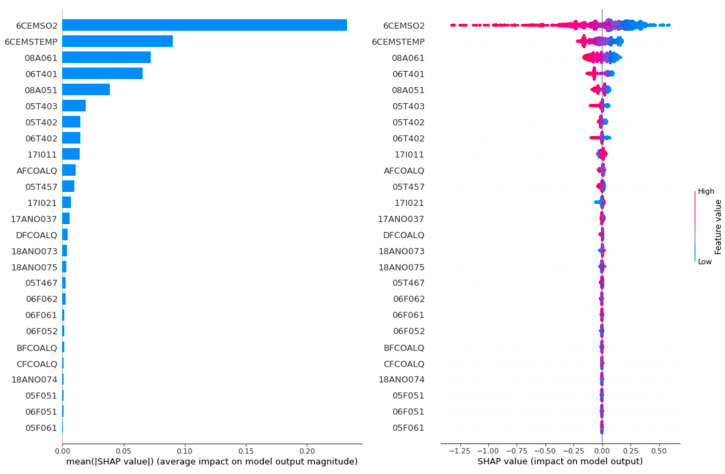
The feature analysis importance diagram and summary diagram of thermal efficiency.

The feature analysis importance diagram and summary diagram of thermal efficiency are shown in Figure 9.

**Figure 10:**
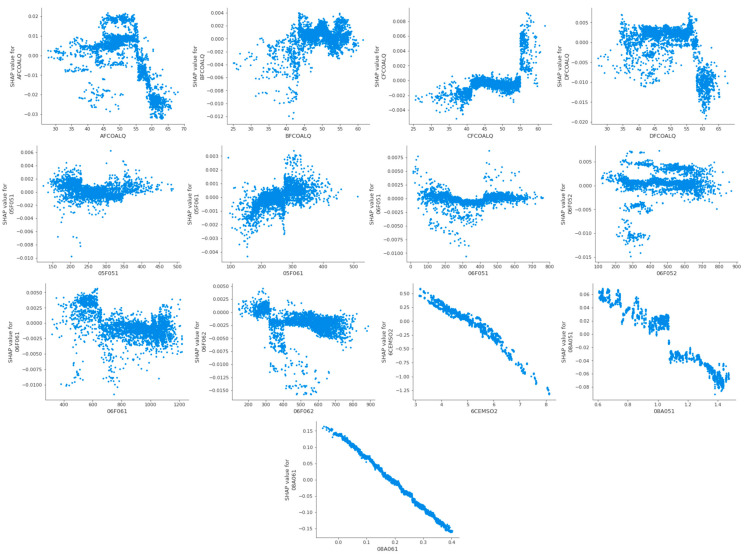
The feature analysis dependency diagram of thermal efficiency.

The feature analysis dependency diagram of thermal efficiency is shown in Figure 10.

**Figure 11:**
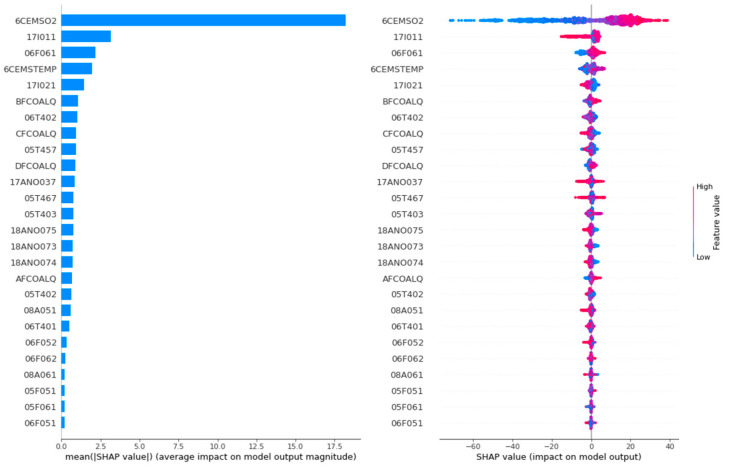
The feature analysis importance diagram and summary diagram of NOx emission concentration.

The feature analysis importance diagram and summary diagram of NOx emission concentration is shown in Figure 11.

**Figure 12:**
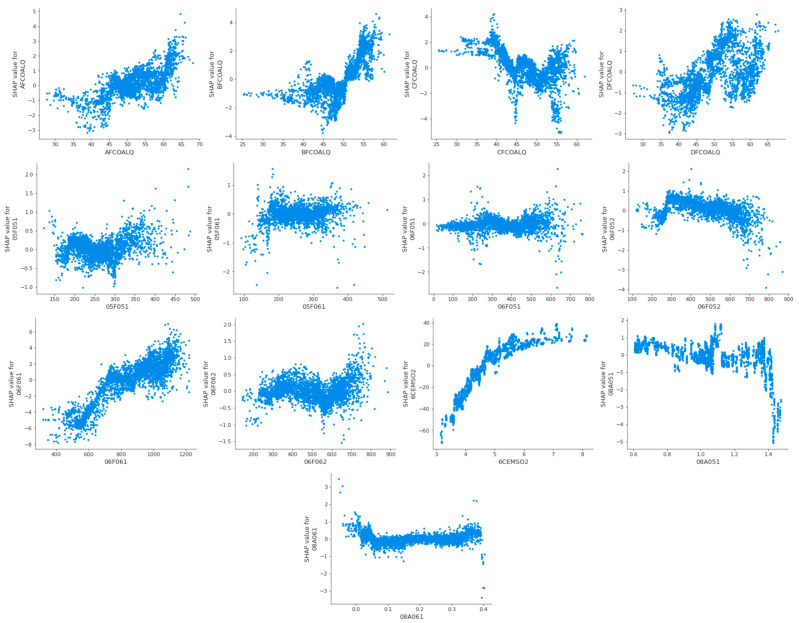
The feature analysis dependency diagram of thermal efficiency.

The feature analysis dependency diagram of thermal efficiency is shown in Figure 12.

In the feature analysis importance diagrams of Figure 9 and Figure 11, the horizontal coordinate represents the Shapley value. The larger the Shapley value, the greater the feature importance. As can be seen in Figure 9, the top five features that have the greatest impact on the thermal efficiency of the CFBB are 6CEMSO2, 6CEMSTEMP, 08A061, 06T401 and 08A051. As can be seen in Figure 11, the top five features that have the greatest effect on the NOx emission concentration of the CFBB are 6CEMSO2, 17I011, 06F061, 6CEMSTEMP and 17I021.

In the feature analysis summary diagrams of Figure 9 and Figure 11, each feature point represents the corresponding feature sample, the change in color from blue to red indicates the change in the value of the feature sample from small to large, and the positive and negative Shapley values indicate the positive and negative effects of the feature sample with the target. In order to explore more visually the positive and negative effects of different values of each feature on the target, the feature analysis dependency diagrams shown in Figure 10 and Figure 12 are more important.

Taking the first subgraph in Figure 10 and Figure 12 as an example, for the feature AFCOALQ, its original value range is [27.67, 68.12]. When it has a positive impact on the thermal efficiency of the CFBB, its value range is [27.67, 60.40], and when it has a negative impact on the NOx emission concentration of the CFBB, its value range is [27.67, 63.86], so it can be obtained that the value range when its impact on thermal efficiency is positive and its impact on NOx emission concentration is negative is [27.67, 56.90]. And the value ranges of the other adjustable parameters of the combustion system of the CFBB are shown in Table 13.

### 4.4. Parameters Optimized by MSMA

In this subsection, the MSMA is applied to optimize the boiler thermal efficiency and NOx emissions concentration based on the established models. The objective function is shown in Equation (24), where X is a vector containing 13 adjustable operation parameters, i.e., AFCOALQ ($x_{1}$), BFCOALQ ($x_{2}$), CFCOALQ ($x_{3}$), DFCOALQ ($x_{4}$), 05F051 ($x_{5}$), 05F061 ($x_{6}$), 06F051 ($x_{7}$), 06F052 ($x_{8}$), 06F061 ($x_{9}$), 06F062 ($x_{10}$), 6CEMSO2 ($x_{11}$), 08A051 ($x_{12}$) and 08A061 ($x_{13}$). $f_{TE}(X)$ and $f_{NOx}(X)$ are the function value of boiler thermal efficiency and NOx emission concentration, respectively. The constraints are shown in Equations (26)–(38), i.e., the range of adjustable operation parameters.

Objective function:

(24) $${min\;f\left(X\right)=f}_{NOx}\left(X\right)-f_{TE}(X)$$

(25) $$X=[x_{1},x_{2},x_{3},x_{4},x_{5},x_{6},x_{7},x_{8},x_{9},x_{10},x_{11},x_{12},x_{13}]$$

Constraints:

(26) $${27.67\leq x}_{1}\leq 56.90$$

(27) $${33.03\leq x}_{2}\leq 51.09$$

(28) $$42.49{\leq x}_{3}\leq 61.93$$

(29) $${33.92\leq x}_{4}\leq 58.11$$

(30) $${151.97\leq x}_{5}\leq 456.13$$

(31) $$91.78{\leq x}_{6}\leq 448.35$$

(32) $${15.05\leq x}_{7}\leq 762.78$$

(33) $${121.21\leq x}_{8}\leq 56.63$$

(34) $$320.80{\leq x}_{9}\leq 1205.40$$

(35) $${144.19\leq x}_{10}\leq 547.852$$

(36) $${3.15\leq x}_{11}\leq 5.14$$

(37) $${0.74\leq x}_{12}\leq 1.07$$

(38) $$0.01{\leq x}_{13}\leq 0.20$$

**Figure 13:**
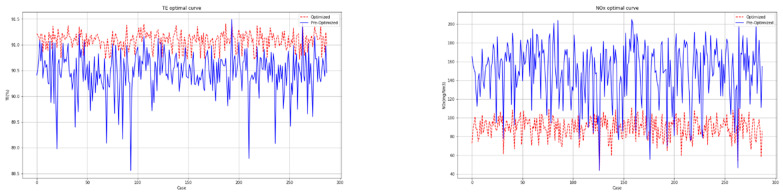
The optimization curves of MSMA for thermal efficiency and NOx emission concentration.

The population size and the maximum number of iterations of the MSMA are set to 40 and 50, respectively. Overall, 10% of the operation data shown in Table 10 were randomly selected to be optimized. The partial optimization results of the MSMA are recorded in Table 13. And the optimization curves of the MSMA for boiler thermal efficiency and NOx emission concentration are shown in Figure 13.

As can be seen in Table 14 and Figure 13, the MSMA can effectively optimize the combustion system of the CFBB, which is able to reduce the NOx emission concentration while ensuring a high thermal efficiency. Among them, the average optimization ratio for thermal efficiency reaches +0.68%, the average optimization ratio for NOx emission concentration reaches −37.55%, and the average optimization time is 6.40 s, which is much less than the time for each interval sampling, so the actual industrial demand can be met.

## 5. Conclusions

This paper designs a new multi-objective optimization framework to solve the boiler combustion optimization problem. The proposed framework has several functions, such as modeling, data correlation analysis and optimization. Actually, the framework integrates the interpretable CatBoost model and a modified slime mould algorithm. The framework is used to improve the thermal efficiency and reduce the NOx emissions of one 330 MW CFBB. The experimental results show that the proposed framework can on average improve the boiler thermal efficiency by 0.68% while reducing the NOx emission concentration by 37.55%, and the optimized adjustment operation parameters can meet the actual industrial demand. In addition, a kind of modified slime mould algorithm is proposed to improve the convergence accuracy of the original SMA. Through the benchmark functions test, the MSMA outperforms the original SMA.

In future research, we are planning to use the proposed framework to solve modeling and optimization problems for different types of complex systems. Moreover, we will further enhance the convergence accuracy of the MSMA through several mechanisms and design more efficient multi-objective algorithms based on the MSMA.

## Figures and Tables

**Table 1:** The symbol explanation of SMA and modified SMA.

Symbol	Explanation
X(t)	The positions of the slime mould individuals at the tth iterations
$X_{best}(t)$	The best position of the slime mould individual at the tth iteration
$X_{A}\left(t\right)$& $X_{B}\left(t\right)$	The randomly selected slime mould individual at the tth iteration
vb	A random value
a	A changing value
P	The total number of iterations
vc	A random value
r	A random value
q	A changing value
f(·)	The output function of the fitness value
$X_{i}$	The position of the ith slime mould individual
S	The optimal fitness value in all iterations
Q	The population size of the slime mould individuals
W	The weight of the slime mould individual
$f_{best}$	The best fitness value at the current iteration
$f_{worst}$	The worst fitness value at the current iteration
SortIndex(·)	The output function of sorted fitness values
d	A random value
$X_{ub}$	The upper bounds of the search space
$X_{lb}$	The lower bounds of the search space
u	The proportion of randomly distributed slime mould individuals
$Z_{i}$	The chaotic sequence
β	A random value
${X_{A}}^{*}$& ${X_{B}}^{*}$	The randomly selected slime mould individual at the tth iteration
$X^{*}$	The elite reverse solution
$X^{**}$	The reverse interpolation solution

**Table 2:** The description of ten benchmark testing functions.

Function	Expression	Range	$f_{min}$
F1	$f_{1}(x)=\sum_{i=1}^{n}\;x_{i}^{2}$	${\left[-100,\;100\right]}^{n}$	0
F2	$f_{2}(x)=\sum_{i=1}^{n}\;\left|x_{i}\right|+\Pi_{i=1}^{n}\left|x_{i}\right|$	${\left[-10,10\right]}^{n}$	0
F3	$f_{3}(x)=\sum_{i=1}^{n}\;{\left(\sum_{j=1}^{i}\;x_{j}\right)}^{2}$	${\left[-100,100\right]}^{n}$	0
F4	$f_{4}(x)=\underset{i}{max}\;\left\{\left|x_{i}\right|,1\leq i\leq n\right\}$	${\left[-10,10\right]}^{n}$	0
F5	$f_{5}(x)=\sum_{i=1}^{n-1}\;\left(100{\left(x_{i+1}-x_{i}^{2}\right)}^{2}+{\left(x_{i}-1\right)}^{2}\right)$	${\left[-30,30\right]}^{n}$	0
F6	$f_{6}(x)=\sum_{i=1}^{n}\;{\left(x_{i}+0.5\right)}^{2}$	${\left[-100,100\right]}^{n}$	0
F7	$f_{7}(x)=\sum_{i=1}^{n}\;ix_{i}^{4}+random[0,1]$	${\left[-1.28,1.28\right]}^{n}$	0
F8	$f_{8}(x)=\sum_{i=1}^{n}\;-x_{i}sin\left(\sqrt{\left|x_{i}\right|}\right)$	${\left[-500,500\right]}^{n}$	−418.9829×n
F9	$f_{9}(x)=\sum_{i=1}^{n}\;\left(x_{1}^{2}-10cos\left(2\pi x_{i}\right)+10\right)$	${\left[-5.12,5.12\right]}^{n}$	0
F10	$f_{10}(x)=-20exp\left(-0.2\sqrt{\frac{1}{n}\sum_{i=1}^{n}\;x_{i}^{2}}\right)-exp\left(\frac{1}{n}\sum_{i=1}^{n}\;cos\left(2\pi x_{i}\right)\right)+20+e$	${\left[-32,32\right]}^{n}$	0

**Table 3:** Parameters setting of six optimization algorithms.

Algorithm	Algorithm Parameters Setting
MSMA	the location update parameter is 0.03, the chaos mapping parameter is 0.7, the proportion of elite individuals is 10%
SMA	the location update parameter is 0.03
BOA	the witching probability is 0.8, the power exponent is 0.1, the sensory modality is 0.1
WOA	the probability parameter of strategy execution is 0.5
SOA	the ratio of flight path to speed is 0.7
SSA	the early warning value is 0.8, the proportion of discoverers is 0.2, the proportion of aware of dangerous sparrows is 0.1

**Table 4:** The comparison metrics for six algorithms on 30 dimensional functions.

Function	Metrics	MSMA	SMA	BOA	WOA	SOA	SSA
F1	mean	2.98 × 10^−310^	2.31 × 10^−242^	5.72 × 10^−10^	3.40 × 10^−22^	1.83 × 10^−200^	1.16 × 10^−12^
	S.D.	0.00	0.00	2.51 × 10^−11^	7.38 × 10^−22^	0.00	6.27 × 10^−12^
	running time	4.01 s	3.87 s	2.37 s	2.48 s	1.13 s	3.07 s
F2	mean	5.12 × 10^−165^	1.94 × 10^−157^	1.11 × 10^−6^	5.13 × 10^−15^	6.85 × 10^−122^	3.00 × 10^−5^
	S.D.	0.00	1.041 × 10^−156^	3.92 × 10^−7^	7.16 × 10^−15^	3.07 × 10^−121^	1.25 × 10^−4^
	running time	4.19 s	4.05 s	2.54 s	2.61 s	1.22 s	3.35 s
F3	mean	4.00 × 10^−265^	6.74 × 10^−196^	5.35 × 10^−10^	7.84 × 10^−3^	3.72 × 10^4^	2.53 × 10^−10^
	S.D.	0.00	0.00	4.13 × 10^−11^	1.98 × 10^−2^	2.90 × 10^4^	1.35 × 10^−9^
	running time	6.53 s	6.16 s	8.10 s	4.52 s	3.05 s	8.80 s
F4	mean	9.69 × 10^−167^	6.06 × 10^−155^	1.55 × 10^−7^	1.45 × 10^−5^	2.23 × 10^−26^	4.29 × 10^−8^
	S.D.	0.00	3.26 × 10^−154^	7.42 × 10^−9^	2.53 × 10^−5^	1.20 × 10^−25^	1.51 × 10^−7^
	running time	4.16 s	4.01 s	2.43 s	2.57 s	1.17 s	3.20 s
F5	mean	2.82 × 10^1^	2.84 × 10^1^	2.89 × 10^1^	2.71 × 10^1^	2.74 × 10^−1^	1.59 × 10^−5^
	S.D.	2.33 × 10^−1^	1.24 × 10^−1^	2.73 × 10^−2^	1.02	5.56 × 10^−1^	3.31 × 10^−5^
	running time	4.45 s	4.29 s	2.58 s	2.69 s	1.27 s	3.48 s
F6	mean	8.29 × 10^−2^	9.47 × 10^−2^	5.79	1.15	3.45 × 10^−2^	9.50 × 10^−8^
	S.D.	3.68 × 10^−2^	4.40 × 10^−2^	5.29 × 10^−1^	5.51 × 10^−1^	3.01 × 10^−1^	1.26 × 10^−7^
	running time	4.64 s	4.52 s	2.62 s	2.80 s	1.28 s	3.55 s
F7	mean	1.35 × 10^−4^	2.11 × 10^−4^	6.73 × 10^−5^	4.85 × 10^−4^	1.17 × 10^−4^	6.18 × 10^−4^
	S.D.	1.23 × 10^−4^	1.91 × 10^−4^	6.17 × 10^−5^	4.66 × 10^−4^	1.08 × 10^−4^	4.38 × 10^−4^
	running time	4.30 s	4.15 s	2.64 s	2.70 s	1.26 s	3.43 s
F8	mean	−1.00 × 10^4^	−8.80 × 10^3^	−2.44 × 10^3^	−7.22 × 10^3^	−1.25 × 10^4^	−7.74 × 10^3^
	S.D.	6.17 × 10^2^	5.61 × 10^2^	4.90 × 10^2^	2.89 × 10^2^	6.89 × 10^1^	2.05 × 10^3^
	running time	4.28 s	4.15 s	2.47 s	2.57 s	1.12 s	3.22 s
F9	mean	0.00	0.00	1.04 × 10^2^	1.51 × 10^−14^	0.00	1.24 × 10^−10^
	S.D.	0.00	0.00	8.03 × 10^1^	4.38 × 10^−14^	0.00	6.65 × 10^−10^
	running time	4.15 s	4.00 s	2.41 s	2.58 s	1.20 s	3.30 s
F10	mean	4.44 × 10^−16^	4.44 × 10^−16^	1.85 × 10^−7^	3.46 × 10^−12^	4.44 × 10^−16^	2.99 × 10^−6^
	S.D.	0.00	0.00	8.74 × 10^−9^	9.37 × 10^−12^	0.00	9.42 × 10^−6^
	running time	4.31 s	4.13 s	2.91 s	2.69 s	1.30 s	3.62 s

**Table 5:** The comparison metrics for six algorithms on 50 dimensional functions.

Function	Metrics	MSMA	SMA	BOA	WOA	SOA	SSA
F1	mean	1.32 × 10^−277^	2.46 × 10^−222^	6.09 × 10^−10^	2.05 × 10^−17^	4.06 × 10^−204^	3.27 × 10^−11^
	S.D.	0.00	0.00	2.87 × 10^−11^	4.84 × 10^−17^	0.00	1.72 × 10^−10^
	running time	6.68 s	6.50 s	3.64 s	4.18 s	1.75 s	5.01 s
F2	mean	2.89 × 10^−147^	2.07 × 10^−139^	1.23 × 10^20^	1.91 × 10^−12^	1.75 × 10^−121^	1.05 × 10^−6^
	S.D.	1.53 × 10^−146^	1.11 × 10^−138^	6.67 × 10^20^	2.22 × 10^−12^	9.02 × 10^−121^	4.18 × 10^−6^
	running time	6.60 s	6.43 s	3.77 s	4.16 s	1.77 s	5.07 s
F3	mean	7.00 × 10^−210^	3.50 × 10^−148^	5.54 × 10^−10^	1.33	1.45 × 10^5^	2.34 × 10^−10^
	S.D.	0.00	1.88 × 10^−147^	3.87 × 10^−11^	3.63	6.87 × 10^4^	1.26 × 10^−9^
	running time	9.79 s	9.26 s	11.98 s	6.86 s	4.51 s	13.29 s
F4	mean	1.25 × 10^−158^	6.62 × 10^−139^	1.63 × 10^−7^	1.97 × 10^−4^	1.99 × 10^−30^	2.04 × 10^−7^
	S.D.	6.33 × 10^−158^	3.56 × 10^−138^	8.79 × 10^−9^	2.32 × 10^−4^	1.06 × 10^−29^	8.90 × 10^−7^
	running time	6.71 s	6.50 s	3.59 s	4.20 s	1.74 s	5.01 s
F5	mean	4.86 × 10^1^	4.85 × 10^1^	4.89 × 10^1^	4.78 × 10^1^	9.32 × 10^−1^	2.08 × 10^−5^
	S.D.	1.27 × 10^−1^	1.49 × 10^−1^	3.49 × 10^2^	7.57 × 10^−1^	2.49	3.03 × 10^−5^
	running time	6.94 s	6.70 s	3.65 s	4.23 s	1.80 s	5.18 s
F6	mean	1.05	1.25	1.07 × 10^1^	3.19	8.93 × 10^−2^	1.91 × 10^−7^
	S.D.	2.98 × 10^−1^	3.30 × 10^−1^	6.71 × 10^−1^	7.21 × 10^−1^	6.86 × 10^−2^	3.65 × 10^−7^
	running time	6.77 s	6.55 s	3.51 s	4.15 s	1.74 s	5.02 s
F7	mean	1.54 × 10^−4^	3.29 × 10^−4^	6.24 × 10^−5^	9.77 × 10^−4^	1.42 × 10^−4^	5.69 × 10^−4^
	S.D.	1.30 × 10^−4^	3.57 × 10^−4^	7.12 × 10^−5^	1.03 × 10^−3^	1.10 × 10^−4^	3.80 × 10^−4^
	running time	6.93 s	6.69 s	3.87 s	4.37 s	1.87 s	5.32 s
F8	mean	−1.59 × 10^4^	−1.36 × 10^4^	−3.22 × 10^3^	−1.19 × 10^4^	−2.09 × 10^4^	−1.64 × 10^4^
	S.D.	6.45 × 10^2^	8.04 × 10^2^	6.77 × 10^2^	4.55 × 10^2^	4.23 × 10^1^	4.80 × 10^3^
	running time	6.89 s	6.70 s	3.71 s	4.21 s	1.68 s	5.08 s
F9	mean	0.00	0.00	1.59 × 10^2^	8.33 × 10^−14^	0.00	8.46 × 10^−8^
	S.D.	0.00	0.00	1.59 × 10^2^	7.58 × 10^−14^	0.00	4.43 × 10^−7^
	running time	6.83 s	6.55 s	3.62 s	4.25 s	1.80 s	5.16 s
F10	mean	4.44 × 10^−16^	4.44 × 10^−16^	1.88 × 10^−7^	4.82 × 10^−10^	4.44 × 10^−16^	9.30 × 10^−8^
	S.D.	0.00	0.00	6.70 × 10^−9^	4.60 × 10^−10^	0.00	3.18 × 10^−7^
	running time	7.60 s	7.33 s	4.60 s	4.77 s	2.10 s	6.07 s

**Table 6:** The comparison metrics for six algorithms on 100 dimensional functions.

Function	Metrics	MSMA	SMA	BOA	WOA	SOA	SSA
F1	mean	1.25 × 10^−249^	2.78 × 10^−193^	6.31 × 10^−10^	1.51 × 10^−13^	4.51 × 10^−208^	6.83 × 10^−12^
	S.D.	0.00	0.00	2.42 × 10^−11^	3.30 × 10^−13^	0.00	3.64 × 10^−11^
	running time	12.82 s	12.49 s	6.47 s	8.15 s	3.18 s	9.46 s
F2	mean	8.63 × 10^−126^	7.83 × 10^−111^	4.82 × 10^50^	6.93 × 10^−10^	1.86 × 10^−120^	1.29 × 10^−6^
	S.D.	4.65 × 10^−125^	4.21 × 10^−110^	2.44 × 10^51^	8.53 × 10^−10^	1.00 × 10^−119^	6.32 × 10^−6^
	running tim × 10	14.0253	13.5653	7.4208	8.8165	3.45 s	10.3113
F3	mean	4.71 × 10^−164^	8.45 × 10^−92^	6.00 × 10^−10^	1.79 × 10^2^	7.00 × 10^5^	1.26 × 10^−6^
	S.D.	0.00	4.55 × 10^−91^	3.30 × 10^−11^	2.23 × 10^2^	3.60 × 10^5^	6.82 × 10^−6^
	running time	21.50 s	20.25 s	25.96 s	15.12 s	9.72 s	29.13 s
F4	mean	4.64 × 10^−153^	1.87 × 10^−142^	1.68 × 10^−7^	2.43 × 10^−3^	3.92 × 10^−32^	6.58 × 10^−9^
	S.D.	2.14 × 10^−152^	1.01 × 10^−141^	6.52 × 10^−9^	2.21 × 10^−3^	2.04 × 10^−31^	2.27 × 10^−8^
	running time	13.03 s	12.64 s	6.54 s	8.22 s	3.20 s	9.58 s
F5	mean	9.87 × 10^1^	9.87 × 10^1^	9.89 × 10^1^	9.78 × 10^1^	4.85 × 10^−1^	2.31 × 10^−5^
	S.D.	1.16 × 10−1	1.14 × 10^−1^	2.46 × 10^−2^	6.45 × 10^−1^	6.17 × 10^−1^	5.55 × 10^−5^
	running time	13.26 s	12.84 s	6.55 s	8.20 s	3.23 s	9.67 s
F6	mean	9.82	1.06 × 10^1^	2.30 × 10^1^	1.03 × 10^1^	1.76 × 10^−1^	1.60 × 10^−7^
	S.D.	1.28	1.20	8.00 × 10^−1^	1.23	1.77 × 10^−1^	2.08 × 10^−7^
	running time	14.65 s	14.17 s	7.16 s	9.14 s	3.57 s	10.61 s
F7	mean	2.41 × 10^−4^	3.69 × 10^−4^	4.88 × 10^−5^	1.10 × 10^−3^	1.58 × 10^−4^	4.35 × 10^−4^
	S.D.	2.08 × 10^−4^	4.09 × 10^−4^	3.56 × 10^−5^	1.50 × 10^−3^	1.35 × 10^−4^	3.43 × 10^−4^
	running time	13.58 s	13.17 s	6.98 s	8.61 s	3.40 s	10.08 s
F8	mean	−2.92 × 10^4^	−2.47 × 10^4^	−4.47 × 10^3^	−2.40 × 10^4^	−4.18 × 10^4^	−3.40 × 10^4^
	S.D.	1.24 × 10^3^	1.31 × 10^3^	9.75 × 10^2^	8.97 × 10^2^	1.85 × 10^2^	9.05 × 10^3^
	running time	14.00 s	13.64 s	7.04 s	8.72 s	3.15 s	10.05 s
F9	mean	0.00	0.00	1.22 × 10^2^	2.48 × 10^−8^	0.00	2.94 × 10^−9^
	S.D.	0.00	0.00	2.74 × 10^2^	1.33 × 10^−7^	0.00	1.58 × 10^−8^
	running time	13.04 s	12.63 s	6.45 s	8.27 s	3.21 s	9.58 s
F10	mean	4.44 × 10^−16^	4.44 × 10^−16^	1.93 × 10^−7^	7.94 × 10^−8^	4.44 × 10^−16^	2.94 × 10^−7^
	S.D.	0.00	0.00	6.85 × 10^−9^	1.70 × 10^−7^	0.00	1.51 × 10^−6^
	running time	13.99 s	13.54 s	7.52 s	8.78 s	3.52 s	10.57 s

**Table 7:** The comparison of model performance for six algorithms on the optimal design of three-bar truss problem.

Metrics	MSMA	SMA	BOA	WOA	SOA	SSA
Mean	2.63 × 10^2^	2.63 × 10^2^	2.64 × 10^2^	2.68 × 10^2^	2.67 × 10^2^	2.63 × 10^2^
Std	2.45 × 10^−3^	3.68 × 10^−3^	5.38 × 10^−1^	4.70	3.95	3.75 × 10^−2^
Min	2.63 × 10^2^	2.63 × 10^2^	2.64 × 10^2^	2.64 × 10^2^	2.63 × 10^2^	2.63 × 10^2^
Max	2.63 × 10^2^	2.63 × 10^2^	2.66 × 10^2^	2.80 × 10^2^	2.76 × 10^2^	2.64 × 10^2^
Time	0.69 s	0.64 s	0.74 s	0.33 s	0.40 s	0.70 s

**Table 8:** The comparison of model performance for six algorithms on the optimal design of tension–compression spring problem.

Metrics	MSMA	SMA	BOA	WOA	SOA	SSA
Mean	1.35 × 10^−2^	1.40 × 10^−2^	8.66 × 10^32^	1.31 × 10^−2^	6.66 × 10^31^	2.33 × 10^32^
Std	8.79 × 10^−4^	2.95 × 10^−3^	3.39 × 10^32^	4.99 × 10^−4^	2.49 × 10^32^	4.22 × 10^32^
Min	1.26 × 10^−2^	1.26 × 10^−2^	1.31 × 10^−2^	1.26 × 10^−2^	1.29 × 10^−2^	1.27 × 10^−2^
Max	1.62 × 10^−2^	2.93 × 10^−2^	1.00 × 10^33^	1.48 × 10^−2^	1.00 × 10^33^	1.00 × 10^33^
Time	0.77 s	0.72 s	0.68 s	0.35 s	0.37 s	0.61 s

**Table 9:** Description of feature symbols.

Symbol	Description
17ANO037	Boiler load
AFCOALQ	Coal feeder feed rate 1
BFCOALQ	Coal feeder feed rate 2
CFCOALQ	Coal feeder feed rate 3
DFCOALQ	Coal feeder feed rate 4
18ANO073	Average bed temperature in the upper part of the dense phase zone of the left side furnace
18ANO074	Average bed temperature in the upper part of the dense phase zone of the furnace chamber
18ANO075	Average bed temperature in the upper part of the dense phase zone of the right side furnace
05F051	Primary air flow rate at the inlet of the left duct burner
05F061	Right side duct burner inlet primary air flow rate
06F051	Total secondary air flow rate on the left side
06F052	Left inner secondary air distribution flow rate
06F061	Total secondary air flow rate on the right side
06F062	Secondary air distribution flow rate in the right side
05T457	Left duct burner inlet primary air temperature
05T467	Right side duct burner inlet primary air temperature
17I011	Limestone powder conveying motor current 1
17I021	Limestone powder conveying motor current 2
6CEMSO2	Flue gas O_2_ concentration
6CEMSTEMP	Flue gas temperature
08A051	Carbon content of fly ash at the inlet of the left side dust collector
08A061	Carbon content of fly ash at the inlet of the right side dust collector
05T402	Primary fan inlet temperature 1
05T403	Primary fan inlet temperature 2
06T401	Secondary fan inlet temperature 1
06T402	Secondary fan inlet temperature 2
NO_X_	NO_X_ emission concentration
TE	Boiler thermal efficiency

**Table 10:** The operation conditions of circulation fluidized bed boiler.

No.	17ANO037	AFCOALQ	BFCOALQ	CFCOALQ	DFCOALQ	18ANO073	18ANO074
1	73.52	37.60	39.17	39.27	37.87	865.03	863.21
2	73.40	39.17	40.41	40.40	39.01	863.65	862.11
3	73.52	39.28	40.41	40.44	39.04	862.89	861.65
…	…	…	…	…	…	…	…
2878	66.30	31.48	47.21	32.74	35.99	855.49	858.57
2879	66.05	31.10	47.11	32.39	35.46	855.84	857.58
2880	65.07	32.17	48.29	33.71	36.94	856.58	856.53
No.	18ANO075	05F051	05F061	06F051	06F052	06F061	06F062
1	861.27	236.65	228.64	23.89	196.35	412.66	188.05
2	860.54	237.11	238.25	107.43	148.38	384.34	216.19
3	860.44	219.26	371.45	188.84	168.03	397.97	211.99
…	…	…	…	…	…	…	…
2878	861.63	181.49	252.21	359.57	120.06	353.54	146.48
2879	859.29	226.35	297.07	190.77	121.21	352.30	170.98
2880	856.41	202.55	304.39	233.81	125.69	347.88	153.15
No.	05T457	05T467	17I011	17I021	6CEMSO2	6CEMSTEMP	08A051
1	269.34	267.38	112.87	101.66	5.55	152.66	0.85
2	269.34	267.38	109.93	101.54	5.55	152.66	0.85
3	269.34	267.38	109.32	97.96	5.55	152.66	0.85
…	…	…	…	…	…	…	…
2878	264.82	261.96	101.77	84.57	7.27	149.11	0.63
2879	264.82	261.96	104.02	84.80	7.27	149.11	0.63
2880	264.82	261.96	100.28	84.61	7.27	149.11	0.63
No.	08A061	05T402	05T403	06T401	06T402	6CEMSNOX	TE
1	0.32	25.65	24.90	23.89	25.73	131.52	90.55
2	0.27	25.65	24.90	23.89	25.73	134.35	90.59
3	0.06	25.65	24.90	23.89	25.73	134.88	90.75
…	…	…	…	…	…	…	…
2878	0.39	24.58	23.28	23.19	23.75	174.25	89.96
2879	0.26	24.58	23.28	23.19	23.75	175.39	90.06
2880	0.13	24.71	23.21	23.22	23.84	174.63	90.13

**Table 11:** Parameter settings of four methods.

Method	Parameters Setting
CatBoost	iterations = 1000, learning_rate = 0.03, max_depth = 6, l2_leaf_reg = 3.
RF	n_estimators = 100, max_depth = None, min_samples_split = 2, min_samples_leaf = 1, max_features = Auto.
SVM	kernel = rbf, gamma = scale, C = 1.0, epsilon = 0.1.
MLP	hidden_size = 100, activation_function = relu, learning_rate = 0.001, iteration = 1000.

**Table 12:** The experimental results of four models on two objectives.

Objective	Dataset	Metrics	Interpretable CatBoost	RF	SVM	MLP
TE	Training data	RMSE	1.85 × 10^−3^	5.75 × 10^−3^	4.66 × 10^−2^	4.96 × 10^−2^
		MAE	1.45 × 10^−3^	3.90 × 10^−3^	3.81 × 10^−2^	3.81 × 10^−2^
		MAPE	2.95 × 10^−3^	8.36 × 10^−3^	6.79 × 10^−2^	6.78 × 10^−2^
		R^2^	99.98%	99.98%	86.53%	85.62%
	Testing data	RMSE	5.27 × 10^−3^	1.53 × 10^−2^	4.72 × 10^−2^	5.08 × 10^−2^
		MAE	3.51 × 10^−3^	1.05 × 10^−2^	3.84 × 10^−2^	3.91 × 10^−2^
		MAPE	7.61 × 10^−3^	2.10 × 10^−2^	6.86 × 10^−2^	6.95 × 10^−2^
		R^2^	99.85%	98.68%	85.78%	84.47%
NOx	Training data	RMSE	1.19 × 10^−2^	1.22 × 10^−2^	5.76 × 10^−2^	7.32 × 10^−2^
		MAE	9.27 × 10^−3^	8.20 × 10^−3^	4.79 × 10^−2^	5.64 × 10^−2^
		MAPE	2.76 × 10^−2^	1.78 × 10^−2^	8.94 × 10^−2^	1.06 × 10^−1^
		R^2^	99.48%	99.45%	84.62%	74.51%
	Testing data	RMSE	2.92 × 10^−2^	3.28 × 10^−2^	6.10 × 10^−2^	7.44 × 10^−2^
		MAE	2.13 × 10^−2^	2.21 × 10^−2^	4.96 × 10^−2^	5.71 × 10^−2^
		MAPE	4.25 × 10^−2^	4.48 × 10^−2^	9.27 × 10^−2^	1.07 × 10^−1^
		R^2^	96.79%	95.84%	82.46%	73.47%

**Table 13:** The value range of adjustable parameters of the combustion system of the CFBB.

Decision Variable	Original Range	Range of Positive Effect on TE	Range of Negative Effect on NOx	Reduced Range
AFCOALQ	[27.67, 68.12]	[27.67, 60.40]	[27.67, 63.86]	[27.67, 56.90]
BFCOALQ	[25.17, 61.44]	[33.03, 61.44]	[25.17, 56.31]	[33.03, 51.09]
CFCOALQ	[25.54, 61.93]	[35.00, 61.93]	[39.59, 61.93]	[42.49, 61.93]
DFCOALQ	[27.23, 67.89]	[33.92, 58.11]	[27.23, 62.84]	[33.92, 58.11]
05F051	[123.59, 487.95]	[123.59, 487.95]	[151.97, 456.13]	[151.97, 456.13]
05F061	[91.78, 513.12]	[91.78, 458.88]	[91.78, 448.35]	[91.78, 448.35]
06F051	[15.05, 762.78]	[15.05, 762.78]	[15.05, 762.78]	[15.05, 762.78]
06F052	[120.06, 869.41]	[121.21, 856.65]	[121.21, 869.41]	[121.21, 856.63]
06F061	[318.67, 1217.79]	[320.80, 1205.40]	[318.67, 1217.79]	[320.80, 1205.40]
06F062	[144.19, 888.01]	[144.19, 728.56]	[144.19, 888.01]	[144.19, 547.852]
6CEMSO2	[3.15, 8.13]	[3.15, 5.49]	[3.15, 5.14]	[3.15, 5.14]
08A051	[0.61, 1.47]	[0.61, 1.07]	[0.74, 1.47]	[0.74, 1.07]
08A061	[−0.01, 0.40]	[−0.01, 0.20]	[0.01, 0.40]	[0.01, 0.20]

**Table 14:** The optimization results of MSMA for thermal efficiency and NOx emission concentration.

No.	TE (Pre-Opt)	TE (Optimized)	TE (Opt-Ratio)	NOx (Pre-Opt)	NOx (Optimized))	NOx (Opt-Ratio)	Time
9	90.32	91.12	+0.89%	139.92	96.59	−30.97%	6.39 s
10	90.40	91.13	+0.81%	138.69	99.60	−28.19%	6.17 s
15	90.70	91.15	+0.49%	134.42	89.80	−33.19%	6.33 s
22	90.62	91.21	+0.65%	149.76	102.44	−31.60%	6.16 s
23	90.72	91.21	+0.54%	150.44	101.99	−32.21%	6.50 s
…	…	…	…	…	…	…	…
2854	90.62	91.06	+0.49%	173.41	102.81	−40.71%	6.36 s
2857	90.71	91.11	+0.43%	173.10	102.95	−40.53%	6.12 s
2861	90.79	91.04	+0.28%	168.90	99.99	−40.80%	6.09 s
2871	90.24	91.04	+0.89%	168.52	94.66	−43.83%	6.07 s
2873	90.23	91.06	+0.93%	170.96	97.38	−43.04%	6.43 s
Average	90.47	91.08	+0.68%	148.95	89.03	−37.55%	6.40 s

## Data Availability

This manuscript does not report data generation or analysis.
